# Improving population health in Germany – lessons of a pilot study to assess health system performance

**DOI:** 10.1093/eurpub/ckac130.010

**Published:** 2022-10-25

**Authors:** K Achstetter, M Blümel, P Hengel, V Schwarzbach, R Busse

**Affiliations:** Department of Health Care Management, Technische Universität Berlin, Berlin, Germany

## Abstract

**Background:**

Improving the population health, both its level and equity, is a major goal of health systems. Health System Performance Assessment (HSPA) is a tool to evaluate the performance of different health system dimensions, e.g., population health, access, efficiency. For the first time, a systematic HSPA was piloted for Germany including the dimension population health.

**Methods:**

The conceptual framework for the German HSPA pilot has been developed in a previous feasibility study. The selection of indicators was based on established indicators used in other HSPA initiatives. Another inclusion criterium was data availability. The ten indicators to measure population health cover e.g., maternal and neonatal health, amenable mortality, infectious diseases, and cancer screening. The indicators are evaluated in terms of their trend over time (2000-2020), in international comparison (e.g., Austria, Denmark, France), and by various equity criteria (e.g., age, gender, region).

**Results:**

Overall, Germany's health system performs moderately regarding population health, especially when compared to selected European countries. While Germany performs very well in terms of incidence rates of infectious diseases, amenable mortality is an area with need for improvements. However, trends over time show improvements of the population health in Germany.

**Conclusions:**

Measuring population health in Germany using ten predefined indicators reveals areas for improvement. Furthermore, subgroup analyses indicate inequities. These results should be considered in further efforts aiming at the improvement of population health in Germany.

**Key messages:**

- Population health in Germany has improved over time.
- However, measuring population health within the German health system identified needs for improvement and inequities.

